# Aspergilloma in combination with adenocarcinoma of the lung

**DOI:** 10.1186/1477-7819-9-27

**Published:** 2011-02-27

**Authors:** Mohamed Smahi, Mounia Serraj, Yassine Ouadnouni, Laila Chbani, Kaoutar Znati, Afaf Amarti

**Affiliations:** 1 Department of thoracic surgery, Hassan II University Hospital of Fez, Morocco; 2 Department of lung disease, Hassan II University Hospital of Fez, Morocco; 3 Laboratory of pathology, Hassan II University Hospital of Fez, Morocco

## Abstract

A 60 year old male with a long standing history of smoking was referred to our department for surgery of aspergilloma in right upper lung lobe diagnosed by computed tomography and confirmed by computed tomography guided needle aspiration biopsy. A lobectomy was performed. Histological study of the surgical specimen revealed a pulmonary adenocarcinoma associated with aspergilloma. By presenting this case we suggest that every case of pulmonary aspergillome should be examined for malignancies, especially in smokers.

## 

In Morocco, pulmonary aspergilloma is most commonly diagnosed in a patient with a healed tuberculous cavity. It rarely affects healthy people with an intact immune response, but those with preexisting structural lung disease, atopy, occupational exposure or impaired immunity are susceptible. Aspergillosis can remain asymptomatic or present with hemoptysis, which can be life-threatening [1]. In this report, we describe a fortuitous discovery of unsuspected lung adenocarcinoma in surgical resection performed for aspergilloma of the right upper lobe.

## Case

**Figure 1:**
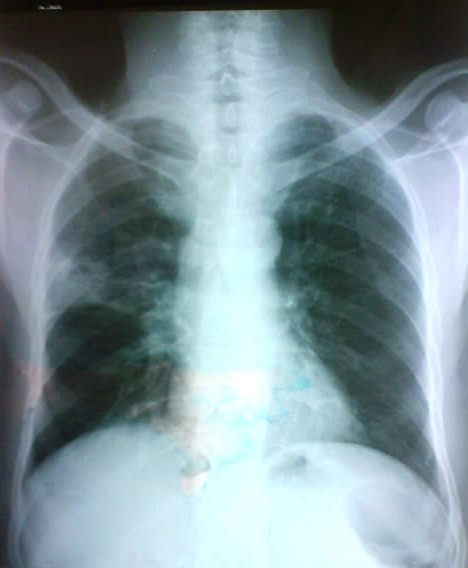
**Cavitary lesion of upper right lobe with "air crescent sign"**.

**Figure 2:**
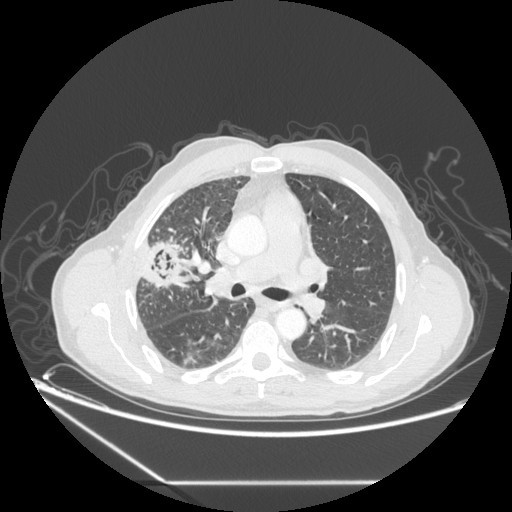
**Cavitated lesion on horseback on the segments of the right upper lobe, with a central heterogeneous rounded density**.

A 60 -year-old man, with social history included a 25 packs/year smoking habit, who was otherwise healthy, presented with history of cough productive with some episodes of small hemoptysis for 7 weeks. There was no history of chest pain, shortness of breath, fever or chills, and he denied any history of weight loss. On physical examination, he appeared healthy with normal findings. Chest radiography revealed a cavitary lesion with "air crescent sign" characteristic of an intracavitary mycetoma (Figure 1), and on CT, there was a cavitary lesion on horseback on the segments of the right upper lung lobe, with a central heterogeneous rounded density, changing position with the patient's movements evoking an aspergilloma (Figure 2). No lesion was detected on fiberoptic bronchoscopy and biopsies were negative. His antifungal serum antibodies were non reactive. CT guided needle aspiration biopsy of the lesion was performed and showed a large number of fungal hyphae of Aspergillus.

**Figure 3:**
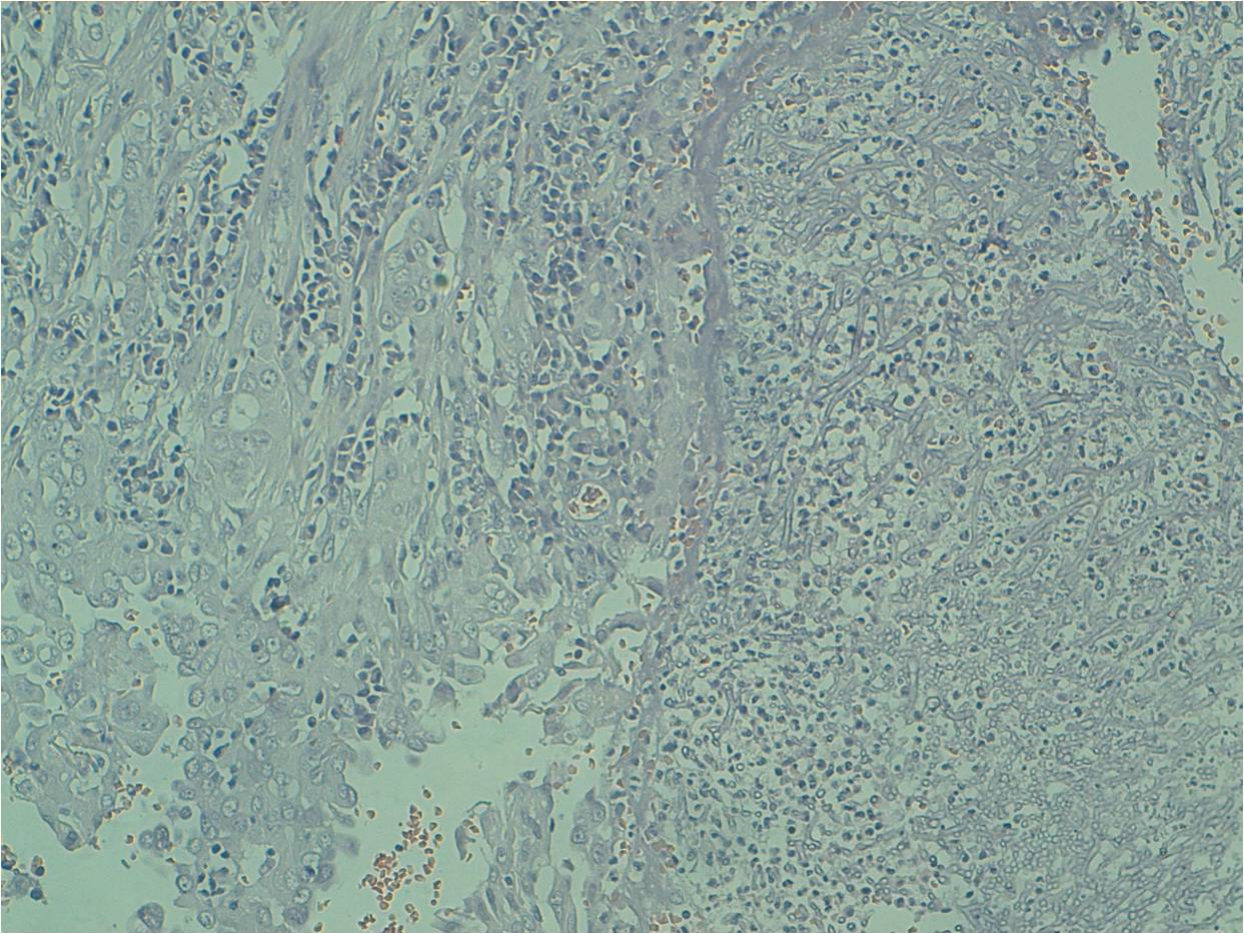
**Histologic appearance from right upper lobectomy demonstrates dichotomously branching hyphae, compatible with Aspergillus associated with adenocarcinoma**. (HES 10x)

Preoperative pulmonary function tests gave normal results. On thoracotomy, a soft mass was palpable in the right upper lobe. Right upper lobectomy was performed. This revealed the presence of an unsuspected 30 mm differentiated and infiltrated lung adenocarcinoma surrounding the 45 mm cavity containing the aspergilloma (Figure 3). Peribronchial and interbronchial nodes were disease free. The patient had an uncomplicated postoperative recovery. The final histological finding confirmed the diagnosis of a T1N0M0 differentiated adenocarcinoma. Chemotherapy or radiotherapy were not considered necessary and it was decided to monitor the progress of the patient with no other treatment.

Twelve months later, the patient is going well with stable X- rays.

## Discussion

Four distinctive patterns of Aspergillus related lung diseases are recognized, as follows: saprophytic colonization, pulmonary aspergilloma, hypersensitivity induced aspergillosis and invasive pulmonary aspergillosis [1].

Pulmonary aspergilloma (PA), or intracavitary fungus ball, is commonly found in cavities such as those seen in cases of sequelar tuberculosis, bronchiectasis, lung cyst and abscess, bullae, pulmonary infarcts, cystic fibrosis, histoplasmosis, sarcoidosis, HIV infection and cavitated squamous cell lung cancer [2]. It is typically caused by Aspergillus fumigatus, although other species may be associated with its formation, usually in the upper lung fields. The diagnosis of PA is usually established radiologically by demonstrating the characteristic appearance of the fungus ball and confirmed by Aspergillosis serology and/or by CT guided needle aspiration biopsy, as in the case here present.

In one study, the prevalence of Aspergillus growth in patients with cavitary or non-cavitary bronchogenic carcinoma was reported as being 14.2% [3], but only a few cases of combined aspergilloma and lung cancer have been reported in the literature [1] because development of an aspergilloma in a cavity associated with a malignant tumor is very unusual.

In the most of the cases, the diagnosis had not been considered preoperatively. The meniscus or air crescent sign is most often associated with benign diseases such as aspergilloma, however, one should remember that carcinoma can be combined [4], especially when patient had an anti fungal agent and the image does not change or continues to increase, when the fungus ball-like shadow is fixed to a thick and irregular wall of the cavity and its position is not altered with the patient's movements [5] and particularly in case of preexisting factor of lung cancer. Frozen section examination of a Wedge excision of aspergilloma performed by video assisted thoracoscopic surgery or thoracotomy must be followed, and when a cancer is combined, a carcinologic surgery and médiastinal lymph node dissection is done.

We suggest that when aspergilloma is found in healthy persons with no risk factors, lung cancer must be ruled out by frozen section of a pulmonary excision of aspergilloma. If combination is confirmed, a carcinologic surgery with mediastinal lymph node dissection must be performed.

## Consent

Written informed consent was obtained from the patient for publication of this case report and accompanying images. A copy of the written consent is available for review by the Editor-in-Chief of this journal.

## Competing interests

The authors declare that they have no competing interests.

## Authors' contributions

MS conceptualized the case study, gathered the data and wrote the manuscript. M Serraj interpreted the data and revised the manuscript. YO acquired the data. LC performed the histopathological evaluation and interpretation of the data. KZ performed the histopathological evaluation and interpretation of the data. AA gave final approval for publication. All authors read and approved the final manuscript.
